# CD271^+^ Subpopulation of Pancreatic Stellate Cells Correlates with Prognosis of Pancreatic Cancer and Is Regulated by Interaction with Cancer Cells

**DOI:** 10.1371/journal.pone.0052682

**Published:** 2012-12-27

**Authors:** Kenji Fujiwara, Kenoki Ohuchida, Kazuhiro Mizumoto, Koji Shindo, Daiki Eguchi, Shingo Kozono, Naoki Ikenaga, Takao Ohtsuka, Shunichi Takahata, Shinichi Aishima, Masao Tanaka

**Affiliations:** 1 Department of Surgery and Oncology, Graduate School of Medical Sciences, Kyushu University, Fukuoka, Japan; 2 Advanced Medical Initiatives, Graduate School of Medical Sciences, Kyushu University, Fukuoka, Japan; 3 Kyushu University Hospital Cancer Center, Fukuoka, Japan; 4 Department of Anatomic Pathology, Graduate School of Medical Sciences, Kyushu University, Fukuoka, Japan; Northwestern University, United States of America

## Abstract

Pancreatic stellate cells (PSCs) play a crucial role in the aggressive behavior of pancreatic cancer. Although heterogeneity of PSCs has been identified, the functional differences remain unclear. We characterized CD271^+^ PSCs in human pancreatic cancer. Immunohistochemistry for CD271 was performed for 31 normal pancreatic tissues and 105 pancreatic ductal adenocarcinomas (PDACs). We performed flow cytometry and quantitative RT-PCR, and assessed CD271 expression in PSCs isolated from pancreatic tissues and the changes in CD271 expression in PSCs cocultured with cancer cells. We also investigated the pattern of CD271 expression in a SCID mouse xenograft model. In the immunohistochemical analyses, the CD271-high staining rates in pancreatic stroma in normal pancreatic tissues and PDACs were 2/31 (6.5%) and 29/105 (27.6%), respectively (p = 0.0069). In PDACs, CD271^+^ stromal cells were frequently observed on the edge rather than the center of the tumors. Stromal CD271 high expression was associated with a good prognosis (p = 0.0040). Flow cytometric analyses demonstrated CD271-positive rates in PSCs were 0–2.1%. Quantitative RT-PCR analyses revealed that CD271 mRNA expression was increased in PSCs after coculture with pancreatic cancer cells. However, the level of CD271 mRNA expression subsequently decreased after the transient increase. Furthermore, CD271 mRNA expression was decreased in PSCs migrating toward pancreatic cancer cells through Matrigel. In the xenograft model, CD271^+^ PSCs were present at tumor margins/periphery and were absent in the tumor core. In conclusion, CD271 was expressed in PSCs around pancreatic tumors, but not in the center of the tumors, and expression decreased after long coculture with pancreatic cancer cells or after movement toward pancreatic cancer cells. These findings suggest that CD271^+^ PSCs appear at the early stage of pancreatic carcinogenesis and that CD271 expression is significantly correlated with a better prognosis in patients with PDAC.

## Introduction

Pancreatic cancer is one of the most lethal cancers, and its 5-year survival rate is only 4% [1]. It is characterized by excessive desmoplasia, which plays a crucial role in its aggressive behavior [2], [3]. Recently, research on cancer biology has focused on cancer–stroma interactions. Interactions between cancer cells and stromal tissues are essential for the development and progression of tumors [4]. Therapies targeting cancer–stroma interactions may represent a new approach for the control of pancreatic cancer.

Pancreatic stellate cells (PSCs) have been identified as the principal source of the excessive extracellular matrix observed in chronic pancreatitis and pancreatic adenocarcinoma [5], [6]. Similar to hepatic stellate cells, an important cell type for extracellular matrix production in hepatic fibrosis, PSCs store fat droplets containing vitamin A within their cytoplasm [7]. PSCs become activated upon stimulation by various autocrine or paracrine factors. They express α-smooth muscle actin (α-SMA) and produce various extracellular matrix proteins [8], [9]. Soluble factors secreted by activated PSCs promote the proliferation, migration, invasion, and survival of pancreatic cancer cells against gemcitabine therapy [10]. Thus, PSCs play an important role in cancer–stroma interactions in pancreatic cancer.

Several reports suggest that stromal cells, such as myofibroblasts and mesenchymal cells, isolated from various human tissues exhibit different phenotypes [11], [12]. We previously reported that CD10^+^ PSCs enhance the progression of pancreatic cancer cells [13]. These observations indicate that PSCs have functional heterogeneity and further suggest that PSCs may contain several other cell subpopulations that individually or synergistically affect the progression of pancreatic cancer. Detailed characterization of the PSCs in pancreatic cancer would help to clarify the mechanism underlying the interactions between cancer cells and stromal cells, and may provide novel targets for stroma-directed therapies.

CD271 (also known as nerve growth factor receptor, NGFR or p75NTR) is a neurotrophin receptor that has been implicated in the paracrine growth regulation of a number of neuronal and non-neuronal tumor types [14], [15]. Recent studies have focused on CD271 because it was identified as a marker of human mesenchymal stem cells [16] and it has been evaluated as an important cancer stem cell marker in melanoma [17]. CD271 might be a marker of a specific functional subpopulation, such as stemness. In the pancreas, CD271 expression in PSCs has been detected [18]. However, CD271 expression rapidly decreased after cell isolation from pancreatic tissues [19]. Therefore, the role of CD271 expression in PSCs remains unclear.

The aim of this study was to identify the specific PSCs that affect the progression of cancer cells by focusing on the expression of CD271. We further assessed the significance of CD271 expression in PSCs.

## Materials and Methods

### Ethics statement

The study was approved by the Ethics Committee of Kyushu University (approval number, 23–64) and conducted according to the Ethical Guidelines for Human Genome/Gene Research enacted by the Japanese Government and the Helsinki Declaration. All the patients provided signed informed consent approving the use of their tissues for unspecified research purposes. For all experiments involving mice, the animals were housed in laminar-flow cabinets under specific pathogen-free conditions in facilities approved by Kyushu University (approval number, A24-112-0).

### Patients and pancreatic tissues

Pancreatic cancer tissues were obtained from 105 patients who underwent pancreatic resection for pancreatic cancer at our institution. The clinicopathologic characteristics of the patients are described in Table S1. Survival was measured from the time of pancreatic resection until death. Prognosis was determined in September 2011. The median overall survival time was 23.5 months (range, 1–114 months). Sixty-two patients died during follow-up. All tissues adjacent to the specimens were evaluated histologically according to the criteria of the World Health Organization. The tumor stage was assessed according to the Union for International Cancer Control (UICC). We also obtained 31 normal pancreas (NP) samples from intact pancreas tissues resected for bile duct cancer, benign solid-pseudopapillary tumor or neuroendocrine tumor for use as control tissues. We further collected 10 pancreatic intraepithelial neoplasia (PanIN) and 39 intraductal papillary mucinous neoplasm (IPMN) samples.

### Immunohistochemical procedures and evaluation

Immunohistochemical staining was performed using a Histofine SAB-PO Kit (Nichirei, Tokyo, Japan). Tissues were sectioned to a thickness of 4 µm and were incubated with mouse monoclonal anti-CD271 antibody (1∶100; Santa Cruz Biotechnology, Santa Cruz, CA) or mouse monoclonal anti-α-SMA antibody (1∶50; Dako, Glostrup, Denmark) overnight at 4°C. Cells were considered to be positively immunostained when the membrane or cytoplasm was stained. We identified activated PSCs based on the cell morphology (spindle-shaped cells) and their identities were confirmed by α-SMA staining. We counted the number of cells in at least 10 fields per section at 200× magnification. To account for the heterogeneity in CD271 expression, the distribution of CD271 immunostaining was evaluated as percentages of the stained cells and scored as follows: 0, 0%; 1, <25%; 2, 26–50%; 3, 51–75%; or 4, 76–100%. Similarly, the staining intensity was scored as follows: 0, no staining; 1, weak staining; 2, moderate staining; or 3, strong staining. Finally, we calculated the staining score by multiplying the percentage score by the intensity score. In the pancreatic cancer cells, we divided the samples into high staining >3 and low staining <2 groups. All slides were evaluated independently by two investigators without any knowledge of the clinical features of each case.

### Cells and culture conditions

Human PSCs were isolated from fresh pancreatic surgical specimens using the outgrowth method in our laboratory [5], [20]. The PSC cell type was confirmed by morphology (stellate-like or spindle-shaped cells) and by immunofluorescence staining for α-SMA and vimentin [10], [13], [20]. PSC1 and PSC3-9 were isolated from pancreatic cancer. PSC2 and PSC10 were isolated from benign pancreatic cyst. PSC11 and PSC12 were isolated from normal pancreatic area of pancreatic cancer tissues. In addition, we evaluated two pancreatic cancer cell lines, SUIT-2 (Health Science Research Resources Bank, Osaka, Japan) and Capan-2 (American Type Culture Collection, Manassas, VA). SK-N-MC, a human neuroepithelioma cell line (American Type Culture Collection), was purchased as a positive control for CD271 expression. Cells at passages 3 to 8 were used for assays. The cells were maintained as previously described [21].

### Immunofluorescence staining and Laser-Scanning confocal microscopy

PSCs (1×10^5^) were plated on glass-bottom dishes (Matsunami, Osaka, Japan) and incubated for 24 hours. In cultures with supernatant, cells were incubated in 10% fetal bovine serum (FBS)/DMEM with supernatant derived from pancreatic cancer cells for 72 hours. The cells were then fixed with methanol, blocked with 3% bovine serum albumin in phosphate-buffered saline solution (PBS), and incubated with a mouse monoclonal anti-α-SMA antibody (1∶50; Dako), a rabbit monoclonal anti-vimentin antibody (1∶50; Cell Signaling Technology, Beverly, MA) and a mouse monoclonal anti-CD271 antibody (1∶50; Santa Cruz Biotechnology) overnight at 4°C. Subsequently, the cells were incubated with Alexa 488-conjugated anti-mouse IgG or 546-conjugated anti-rabbit IgG (Molecular Probes, Eugene, OR) for 1 hour. Nuclear DNA was counterstained with 4′,6-diamidino-2-phenylindole (0.05 µg/mL). A laser-scanning confocal fluorescent microscope (A1R; Nicon, Tokyo, Japan) was used, and images were managed using NIS-Elements software (Nikon).

### Flow cytometry analysis

Cultured cells were obtained from subconfluent monolayer cultures and incubated with a fluorescein isothiocyanate (FITC)-conjugated anti-CD271 antibody (Miltenyi Biotec, Auburn, CA). Mouse IgG1 K Isotype Control FITC (Miltenyi Biotec) was used as a negative control. The labeled cells were analyzed by flow cytometry using a FACS Calibur (Becton Dickson and Company, Franklin Lakes, NJ).

### Real-time quantitative RT-PCR (qRT-PCR)

Total RNA was extracted from cultured cells using a High Pure RNA Isolation Kit (Roche Diagnostics, Mannheim, Germany) and DNase I (Roche Diagnostics). Real-time qRT-PCR was performed using a QuantiTect SYBR Green Reverse Transcription-PCR Kit (Qiagen, Tokyo, Japan) and a Chromo4 Real-Time PCR Detection System (Bio-Rad Laboratories, Hercules, CA). Primers for CD271 and β-actin were purchased from Takara Bio Inc. (Tokyo, Japan). The sequences of the oligonucleotide primers used in this study were as follows: CD271, 5′-TCAGTGGCATGGCTCCAGTC-3′ (forward) and 5′-GCAGTATCCAGTCTCAGCCCAAG-3′ (reverse); β-actin, 5′-TGGCACCCAGCACAATGAA-3′ (forward) and 5′-CTAAGTCATAGTCCGCCTAGAAGCA-3′ (reverse). Each reaction mixture was initially incubated at 50°C for 30 min to allow reverse transcription. PCR amplification was then initiated by incubation at 95°C for 15 min to activate the polymerase, followed by 40 cycles of 95°C for 5 s, 60°C for 20 s, and 72°C for 30 s. The expression levels of genes were calculated using a standard curve constructed using total RNA from SK-N-MC cells. The expression levels were normalized to the β-actin expression levels as an internal control, and expressed as the ratio of expression of the target gene to that of β-actin. All samples were run in triplicate. No detectable PCR products were amplified without prior reverse transcription. The accuracy and integrity of the PCR products were confirmed using an Agilent 2100 Bioanalyzer (Agilent Technologies Inc., Palo Alto, CA).

### In vitro coculture system

*In vitro* cocultures were performed using 6-well cell culture insert companion plates and 3.0-µm cell culture inserts (Becton Dickinson Labware, Franklin Lakes, NJ) as previously described [22]. SUIT-2 and Capan-2 cells (5×10^5^) were seeded separately into the upper chambers at 24 hours after seeding two types of PSCs (5×10^5^) into the lower chambers.

### Culture with a cancer cell-derived supernatant

SUIT-2 and Capan-2 cells were cultured under FBS-free conditions for 48 hours. Thereafter, we collected the supernatants from the cancer cells and adjusted them to 10% FBS. The cancer cell-derived supernatants were then added to previously seeded PSCs for subsequent culture.

### Functional separation by Matrigel invasion assay

Six-well cell culture insert companion plates and 8.0-µm cell culture inserts (Becton Dickinson Labware) were coated with Matrigel (150 µg/well; BD Biosciences, Bedford, MA). PSCs (5×10^5^ cells/2 mL) were seeded into the upper chambers. Previously, cancer cells (5×10^5^) were seeded into the lower chambers. Thereafter, the cells were cultured for 72 hours. Subsequently, we removed the cells from both sides of the upper chambers using trypsin. After two centrifugations, we extracted mRNA from the cells. Each experiment was carried out in triplicate wells, and independent experiments were repeated three times.

### In vivo experiments

SUIT-2 pancreatic cancer cells and two types of PSCs were used for *in vivo* experiments. Cells were divided into three groups, comprising SUIT-2 cells alone, SUIT-2 cells with PSC1 cells, and SUIT-2 cells with PSC2 cells. SUIT-2 cells (5×10^5^) and PSCs (5×10^5^) suspended in 100 µL of PBS were cotransplanted into the pancreatic tail of 6-week-old female SCID mice (FOX CHASE SCID®, C.B-17/lcr-scid/scidJcl; Clea Japan Inc., Tokyo, Japan). Six mice were used in each group. The tumors were resected on days 8, 15, and 22 after implantation. The tissues were fixed in 10% neutral-buffered formalin and embedded in paraffin. Four µm tissue sections were stained with a mouse monoclonal anti-α-SMA antibody (Dako) and a rabbit monoclonal anti-CD271 antibody (Millipore, Billerica, MA).

### Statistical analysis

Values are expressed as means ± SD. Comparisons between two groups were performed using Student's *t*-test. Statistical significance was defined as values of p<0.05. A χ^2^ test was used to analyze the correlations between CD271 expression and the clinicopathologic characteristics observed in the immunohistochemical analyses. Survival analyses were undertaken using the Kaplan–Meier method, and the curves were compared using the log-rank test. To evaluate independent prognostic factors associated with survival, a multivariate Cox proportional-hazards regression analysis was used, with CD271 expression, pT category, lymph node metastasis, UICC stage, perilymphatic invasion, perivascular invasion, and pathological margin as covariates. All statistical analyses were performed using JMP 8.0 software (SAS Institute, Cary, NC).

## Results

### Stromal cells express CD271 in pancreatic ductal adenocarcinoma (PDAC)

**Figure 1:**
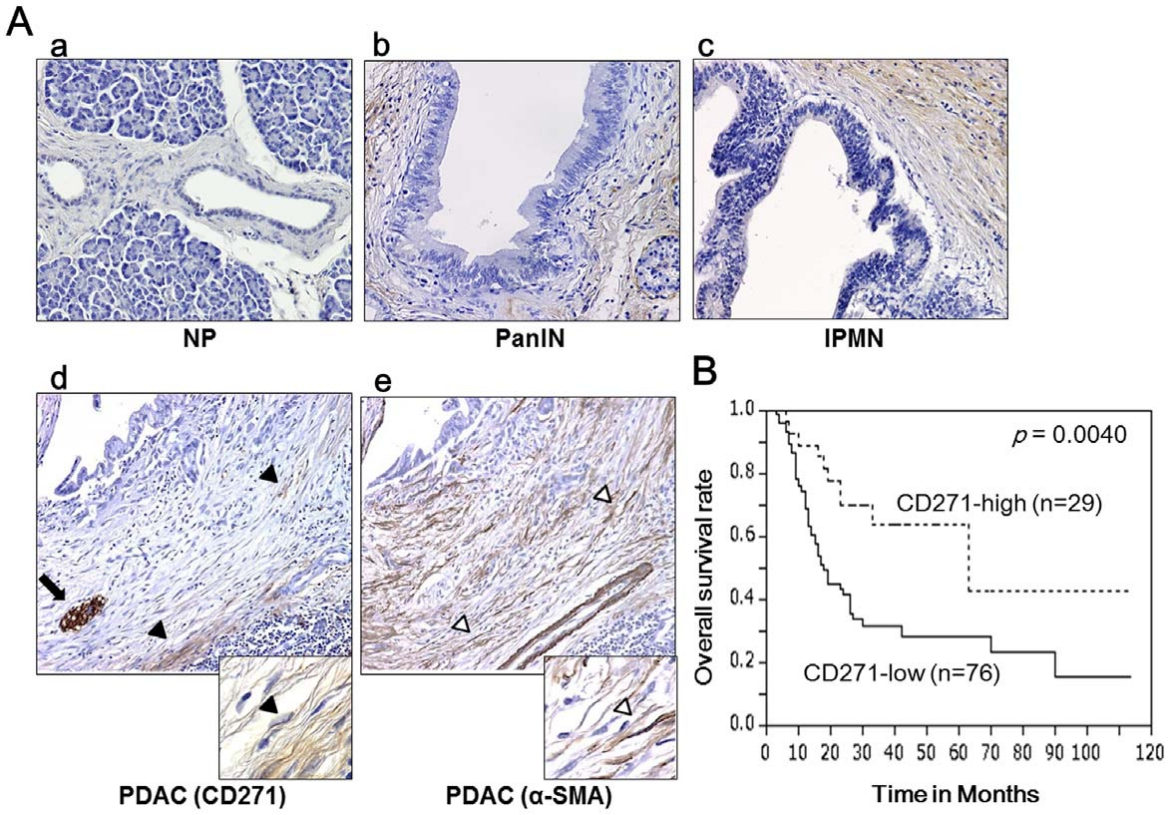
Characterization of stromal CD271 expression in pancreatic cancer. (A) Immunohistochemical staining for CD271 in pancreatic tissues. (A-a) In normal pancreas (NP), stromal cells rarely stain positive for CD271. (A-b, -c) Stromal cells are strongly stained around pancreatic intraepithelial neoplasia (PanIN) (b) and intraductal papillary mucinous neoplasm (IPMN) (c). (A-d) In pancreatic ductal adenocarcinomas (PDAC), stromal cells are partly stained, and CD271^+^ stromal cells are not adjacent to pancreatic cancer cells. Black arrowheads and arrows indicate CD271^+^ cells and nerves as positive control in the serial sections. (A-e) α-smooth muscle actin (SMA) is expressed most stromal cells around cancer cells and neoplastic tubules. White arrowheads indicate α-SMA cells in the serial sections. Original magnification: 200×, insets: 600×. (B) Kaplan–Meier survival analysis for CD271 high expression in the stroma of PDAC. Stromal CD271 high expression is associated with a good prognosis (p = 0.0040).

To evaluate CD271 expression in pancreatic tissues, we performed immunohistochemistry for CD271. Consistent with a previous report [23], we observed strong staining of nerves as a positive control. The epithelial cells of normal pancreatic ducts showed no expression of CD271, and PDAC cells were unstained. In normal pancreas (NP), stromal cells around the pancreatic duct were rarely stained (Figure 1A-a). However, we observed that stromal cells were strongly stained around pancreatic intraepithelial neoplasia (PanIN) (Figure 1A-b) and intraductal papillary mucinous neoplasm (IPMN) (Figure 1A-c). In PDAC tumors, stromal cells around pancreatic cancer cells were partly stained. However, CD271^+^ stromal cells were not adjacent to cancer cells in PDAC, and were strongly stained at the edge rather than the center of the tumors (Figure 1A-d). CD271 high staining in stromal cells was present in 6.5% (2/31) of NP, 30.0% (3/10) of PanIN, 27.6% (29/105) of PDAC and 84.6% (33/39) of IPMN. The CD271 high staining rates were significantly higher in stromal cells of IPMN (p<0.0001) and PDAC (p = 0.0069) than those in stromal cells of NP (Table 1).

**Table 1:** Stromal CD271 high staining rates in surgical pancreatic tissues.

Diagnosis	High staining rates	*p* value
NP	6.5% (2/31)	-
PanIN	30.0% (3/10)	0.0669
PDAC	27.6% (29/105)	0.0069
IPMN	84.6% (33/39)	<0.0001

NP: normal pancreas; PanIN: pancreatic intraepithelial neoplasia; PDAC: pancreatic ductal adenocarcinoma; IPMN: intraductal papillary mucinous neoplasm. Comparisons between NP and the three tumor groups were determined.

### Stromal CD271 expression independently correlated with good prognosis

We evaluated correlations between stromal CD271 expression and the clinicopathologic factors of PDAC. Although differences did not reach statistical significance, pT1/pT2, no lymph node metastasis, UICC stage I, no perilymphatic invasion, no perivascular invasion, and pathological margin/negative were observed more frequently in the stromal CD271high staining group than in the stromal CD271 low staining group (Table 2). Interestingly, stromal CD271 expression was associated with a good prognosis in pancreatic cancer (p = 0.0040) (Figure 1B). The median survival times for CD271 high staining and CD271 low staining patients were 62 and 18.5 months, respectively. Next, we performed a multivariate survival analysis based on the Cox proportional hazard model for all parameters found to be significant by univariate analyses, including stromal CD271 expression, pT category, lymph node metastasis, UICC stage, perilymphatic invasion, perivascular invasion, and pathologic margin positivity (Table S2). Stromal CD271 expression was found to be an independent prognostic marker in pancreatic cancer patients, and the relative risk of stromal CD271 expression was 0.495 (Table 3).

**Table 2:** Relationships between stromal CD271 expression and clinicopathologic factors.

		CD271 low staining	CD271 high staining	
Characteristics		n = 76 (72.4%)	n = 29 (27.6%)	*p* Value
Age	<65	35 (46.1)	16 (55.2)	0.403
	≥65	41 (54.0)	13 (44.8)	
pT category	pT1/pT2	7 (9.21)	7 (24.1)	0.055
	pT3/pT4	69 (90.8)	22 (75.9)	
Histologic grade	G1	9 (11.8)	4 (13.8)	0.564
	G2	24 (31.6)	13 (44.8)	
	G3	39 (51..3)	11 (37.9)	
	others	4 (5.3)	1 (3.5)	
Lymph node metastasis	No	17 (22.4)	12 (41.4)	0.057
	Yes	59 (77.6)	17 (58.6)	
UICC stage	I	5 (6.58)	6 (20.7)	0.128
	II	69 (90.8)	22 (75.9)	
	III/IV	2 (2.63)	1 (3.45)	
Perilymphatic invasion	No	18 (23.7)	12 (41.4)	0.079
	Yes	58 (76.3)	17 (58.6)	
Perivascular invasion	No	28 (36.8)	16 (55.2)	0.09
	Yes	48 (63.2)	13 (44.8)	
Perineural invasion	No	14 (18.4)	5 (17.2)	0.888
	Yes	62 (81.6)	24 (82.8)	
Pathological margin	Negative	50 (65.8)	25 (86.2)	0.082
	Positive	26 (34.2)	4 (13.8)	

UICC, Union for International Cancer Control.

**Table 3:** Multivariate survival analysis (Cox regression model) of conventional prognostic factors and stromal CD271 expression.

	Relative risk	95% Confidence interval	*p* value
CD271 positivity	0.495	0.231–0.962	0.0349
pT category	7.621	1.179–28.117	0.0361
Lymph node metastasis	1.864	0.860–4.658	0.1194
UICC stage	-	-	0.0229
Perilymphatic invasion	1.188	0.509–1.966	0.6751
Perivascular invasion	1.908	1.074–3.717	0.0276
Pathological margin	6.433	0.668–62.02	0.0006

The relative risk of the UICC stage is not shown because it is comprised of two parameters.

UICC, Union for International Cancer Control.

### CD271 stromal cells are a subpopulation of activated PSCs

To characterize the CD271^+^ stromal cells, we performed immunohistochemistry for α-SMA, a marker for activated PSCs, on serial PDAC sections. We found that α-SMA was expressed in most stromal cells around cancer cells and neoplastic tubules (Figure 1A-e). CD271^+^ stromal cells were restricted to areas with strong α-SMA expression. These findings indicate that CD271^+^ stromal cells are a subpopulation of activated PSCs.

### CD271 expression in human PSCs

**Figure 2:**
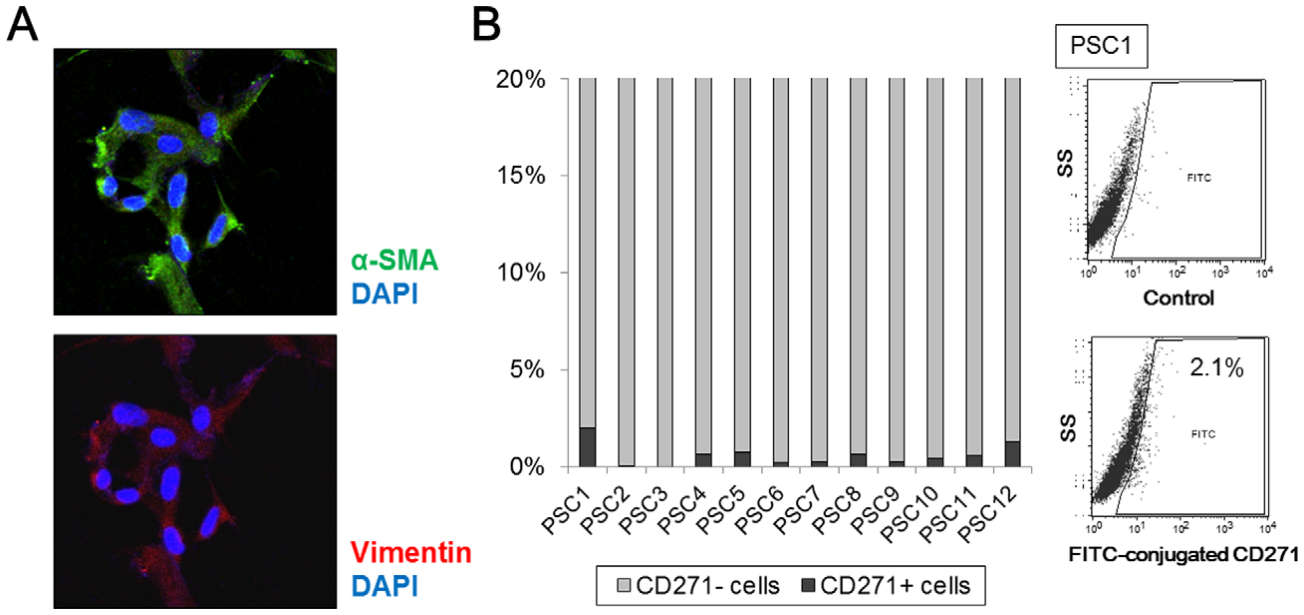
Analyses of CD271 expression in human pancreatic stellate cells (PSCs) isolated from pancreatic tissues. (A) Representative microphotograph of immunofluorescence staining for α-smooth muscle actin (SMA) (green) and vimentin (red) in PSCs. Nuclei were counterstained with 4′,6-diamidino-2-phenylindole (blue). PSCs show a stellate-like or spindle-shaped morphology and express α-SMA. Original magnification: 200×. (B) The positive rates of CD271 expression in PSCs are 0.0–2.1% by flow cytometric analyses. Representative flow cytometric images of CD271 in activated PSCs are also shown (right).

We isolated five primary cultures of PSCs from fresh human pancreatic tissues, and their identity was confirmed by immunofluorescence staining. PSCs were stellate-like or spindle-shaped and expressed α-SMA and vimentin (Figure 2A). In these immunofluorescence analyses, we did not detect CD271 expression in PSCs (data not shown). Next, we evaluated CD271 expression in 12 primary cultures of human PSCs using flow cytometry, and found CD271-positive rates of 0.0–2.1% in PSCs (Figure 2B).

### CD271^+^ PSCs are increased through cancer–stroma interactions

**Figure 3:**
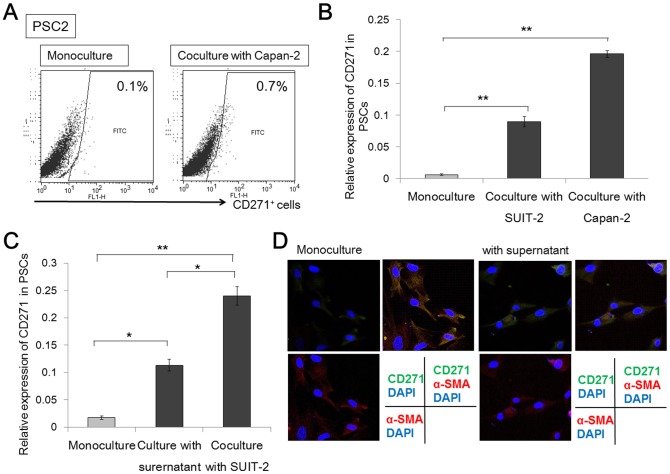
CD271^+^ pancreatic stellate cells (PSCs) are increased through cancer–stroma interactions. (A) In flow cytometry analyses, the percentage of CD271^+^ cells is higher in coculture than in monoculture (0.7–0.8% vs. 0.1%). (B) The levels of CD271 mRNA expression in two cultures of PSCs are significantly higher in cocultures with pancreatic cancer cells than in monocultures (p<0.001). (C) The level of CD271 mRNA expression is higher in cultures with a cancer cell-derived supernatant than in monocultures (p = 0.0053). (D) Representative microphotograph of immunofluorescence staining for CD271 (green) and α-smooth muscle actin (SMA) (red) in PSCs. Nuclei were counterstained with 4′,6-diamidino-2-phenylindole (blue). CD271 in PSCs was slightly expressed in cultures with cancer cell-derived supernatant, although it was difficult to detect CD271 expression in monocultures. Original magnification: 200×. *p<0.01; **p<0.001.

We investigated the effects of PSCs on the phenotype of cancer cells using a coculture system. We used two pancreatic cancer cell lines, SUIT-2 and Capan-2, and two primary cultures of human PSCs isolated from PDAC and benign pancreatic cysts. After monoculture or coculture with pancreatic cancer cells for 72 hours, we evaluated CD271 expression in PSCs using flow cytometry and real-time qRT-PCR. By flow cytometry analyses, we observed the percentage of CD271^+^ cells was higher in coculture than in monoculture (0.7% vs. 0.1%) (Figure 3A). By real-time qRT-PCR analyses, the level of CD271 mRNA expression in two cultures of PSCs was significantly higher in coculture with pancreatic cancer cells than in the monoculture (p<0.001) (Figure 3B). Next, we collected supernatant from SUIT-2 pancreatic cancer cells, and added it to two primary cultures of PSCs. We compared the levels of CD271 mRNA expression among monocultures, cultures with the cancer cell-derived supernatant, and cocultures with pancreatic cancer cells for 72 hours. The level of CD271 mRNA expression was highest in cocultures with pancreatic cancer cells. In addition, the level of CD271 mRNA expression was higher in cultures with the cancer cell-derived supernatant than in monocultures (p = 0.0053) (Figure 3C). By immunofluorescence analyses, we detected CD271 expression in PSCs was slightly increased in culture with the cancer cell-derived supernatant in contrast to monocultures (Figure 3D).

### CD271 expression decreases after the transient increase in expression when cocultured with pancreatic cancer cells

**Figure 4:**
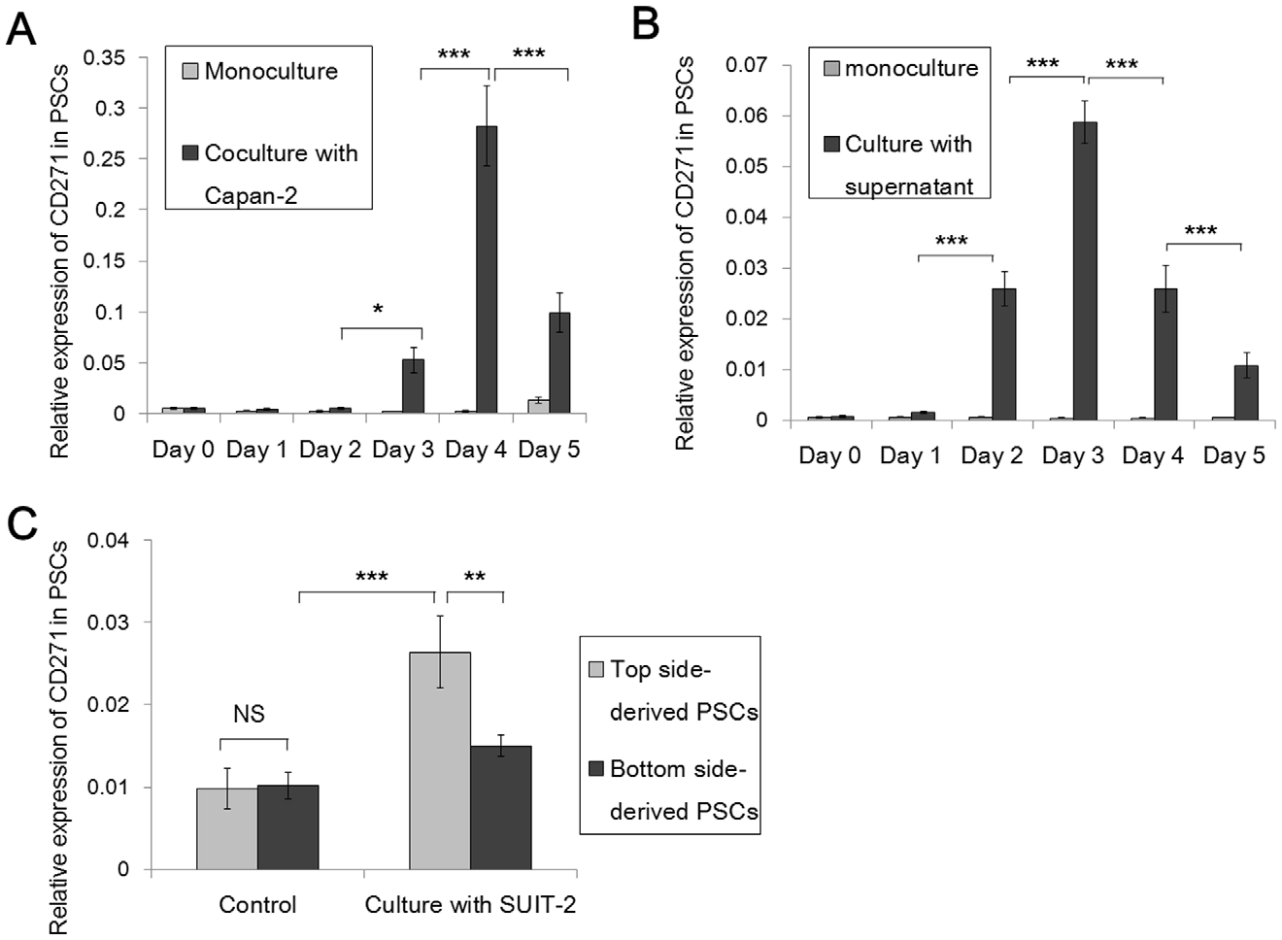
CD271 expression in pancreatic stellate cells (PSCs) decreases after long-term interactions with pancreatic cancer cells. (A) Real-time qRT-PCR analyses showed that the levels of CD271 mRNA expression in PSCs in coculture with pancreatic cancer cells start to increase on day 3 (p = 0.0249), are highest on day 4 (p<0.0001), and then decrease the following day after the peak expression (p<0.0001). (B) In cultures with addition of cancer cell-derived supernatant, the level of CD271 mRNA expression started to increase at day 2 (p<0.0001) and then decreased on day 4 (p<0.0001). (C) Evaluation of the differences in CD271 expression depending on the function of PSCs by real-time qRT-PCR. The level of CD271 mRNA expression in top side-derived PSCs, which did not move toward cancer cells through Matrigel and remained on upper chambers, is lower than that in bottom side-derived PSCs, which did move toward pancreatic cancer cells through Matrigel (p<0.01). *p<0.05; **p<0.01; ***p<0.001; NS, not significant.

We used a coculture system consisting of two pancreatic cancer cell lines, SUIT-2 and Capan-2, and two primary cultures of human PSCs to evaluate the time-dependent changes in CD271 mRNA expression. After monoculture or coculture of PSCs with pancreatic cancer cells for 1 to 5 days, we evaluated the level of CD271 mRNA expression in PSCs. In monocultures, the expression of CD271 mRNA in PSCs did not change. However, in cocultures with pancreatic cancer cells, the level of CD271 mRNA expression increased on day 3 (p = 0.0249) and peaked at day 4 (p<0.0001). Interestingly, the levels of CD271 mRNA expression decreased the day after peak expression (p<0.0001) (Figure 4A). Next, we collected supernatant from Capan-2 pancreatic cancer cells, added it to two primary cultures of PSCs and evaluated the time-dependent changes in CD271 mRNA expression. In cultures with supernatant, the level of CD271 mRNA expression increased on day 2 (p<0.0001) and then decreased on day 4 (p<0.0001) (Figure 4B). Similar to coculture with pancreatic cancer cells, CD271 expression decreased after the transient increase in expression when cocultured with supernatant.

### CD271 expression is decreased in PSCs migrating through Matrigel toward pancreatic cancer cells

Next, we evaluated CD271 expression in PSCs after functional separation using Matrigel invasion assay. In this culture system involving upper chambers coated with Matrigel, we evaluated SUIT-2 pancreatic cancer cells and two primary cultures of human PSCs. PSCs in the upper chambers were incubated for 72 hours in culture with pancreatic cancer cells in the lower chambers, or as controls without pancreatic cancer cells in the lower chambers. PSCs were collected from the top and bottom sides of the upper chambers separately. The top side-derived PSCs were cells that did not migrate toward the cancer cells through the Matrigel and remained in the upper chambers. The bottom side-derived PSCs were cells that migrated toward the pancreatic cancer cells through Matrigel and collected on the bottom side of the upper chambers. We then analyzed CD271 mRNA expression levels in the top or bottom side-derived PSCs to evaluate the functional differences between CD271^+^ and CD271^−^ PSCs. The level of CD271 mRNA expression was highest in top side-derived PSCs after culture with pancreatic cancer cells (p<0.001). Interestingly, the level of CD271 mRNA expression in the bottom side-derived PSCs migrating toward the pancreatic cancer cells was lower than that in the top side-derived PSCs that did not move toward cancer cells (p<0.01). In controls, there were no differences in CD271 mRNA expression levels between top and bottom side-derived PSCs, and these levels were low compared with the levels of CD271 mRNA expression in cultures with pancreatic cancer cells (Figure 4C).

### CD271^+^ PSCs are present in tumor margins/periphery and are absent in the tumor core

**Figure 5:**
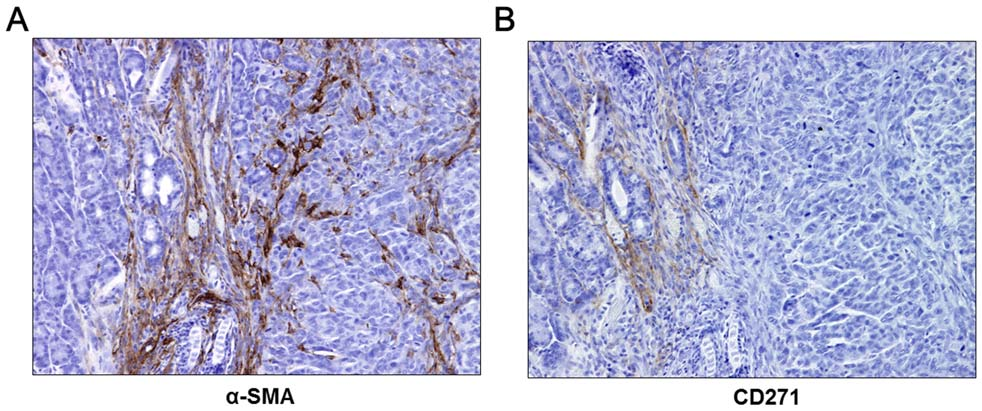
Expression of α-smooth muscle actin (SMA)^+^ and CD271^+^ stromal cells during *in vivo* tumor growth of pancreatic cancer. (A) α-SMA^+^ stromal cells, as activated PSCs, exist in the xenograft tumor and normal pancreas (NP) areas (left side) around the tumor (right side). (B) Few CD271^+^ stromal cells were present in the NP area (left side) around the xenograft tumor, and were absent in the tumor core (right side). Original magnification: 200×.

To evaluate the localization of CD271^+^ PSCs *in vivo*, we transplanted SUIT-2 pancreatic cancer cells into the pancreatic tail of SCID mice, or cotransplanted them with two primary cultures of human PSCs. We euthanized the mice on days 8, 15, or 22, and evaluated the localization of CD271^+^ PSCs in the xenograft tumors. Stromal CD271 expression was detected at all days after transplantation, regardless of transplantation or cotransplantation. Stromal CD271 high staining rates were 50% (3/6), 60% (3/5), and 67% (4/6) on days 8, 15, and 22, respectively. In every case, we detected CD271 expression on nerve bundles as a positive control, and found that pancreatic cancer cells never expressed CD271. We confirmed the presence of activated PSCs by a spindle-shaped morphology and by α-SMA staining. Activated PSCs existed in the xenograft tumors and normal pancreatic areas around the tumors (Figure 5A). However, CD271^+^ PSCs only existed in the normal pancreatic areas around the xenograft tumors, and were absent in the tumor core (Figure 5B).

## Discussion

Previously, Trim et al., [18] reported that CD271 was expressed in PSCs. Haas et al., [19] also reported that CD271 was slightly expressed in PSCs and that the level of CD271 mRNA expression rapidly decreased in primary rat PSCs during culture. We found that CD271^+^ PSCs existed in surgical pancreatic tissues using immunohistochemistry. However, qRT-PCR and flow cytometry analyses revealed that CD271 expression in PSCs isolated from pancreatic tissues was very weak. These findings suggest that CD271 expression in primary human PSCs also rapidly decreased during culture.

We found that CD271 mRNA expression was transiently increased in PSCs cocultured with pancreatic cancer cells, suggesting that pancreatic cancer cells enhance CD271 expression in PSCs. However, the present immunohistochemical analyses revealed that CD271^+^ PSCs also existed around PanIN and IPMN, precancerous lesions. Furthermore, Trim et al., [18] reported that CD271^+^ PSCs were found in tissues from chronic pancreatitis patients. These observations suggest the appearance of CD271^+^ PSCs is not specific to malignant diseases, and is related to desmoplasia. Haas et al., [19] reported that CD271 mRNA expression was enhanced in PSCs by transforming growth factor beta (TGFβ), a strong profibrogenic activator of PSCs. Addition of TGFβ to PSCs slightly increased the level of CD271 mRNA expression in PSCs, but without a significant difference (data not shown). Although TGFβ secreted from pancreatic cancer cells may be related to CD271 expression in PSCs, it is possible that other soluble factors also affect CD271 expression.

Interestingly, CD271^+^ stromal cells were not adjacent to pancreatic cancer cells in PDAC as observed by immunohistochemical analyses. *In vivo*, CD271 expression decreased after the transient increase in expression in PSCs cocultured with pancreatic cancer cells. These findings suggest that CD271 expression is decreased in PSCs following long-term interactions with pancreatic cancer cells. In pancreatic cancer tissues, CD271 high staining cases have a better prognosis than CD271 low staining cases. Progression of PDAC may cause the decrease of CD271 positive rate of PSCs due to the long-term influences of pancreatic cancer cells.

The functional role of CD271^+^ PSCs remains unclear. Previous reports identified CD271 as a marker of activated PSCs [18], [19]. However, CD271 expression in PSCs rapidly decreased during primary culture [19], even though α-SMA expression in PSCs remained after several passages of primary cultures. Recently, several articles have reported that normal fibroblasts, which prevented tumor growth and invasiveness, became reprogrammed by unknown mechanisms to co-evolve with epithelial tumor cells and provide an environment conducive to tumor initiation and progression [24]–[28]. CD271 expression may be a marker for such a reprogramming stage, suggesting that CD271 expression is a temporary marker of PSCs in the early stages of interaction with pancreatic cancer cells. Previously, we reported that CD10^+^ PSCs enhanced the malignant progression of pancreatic cancer [13]. CD10^+^ PSCs existed near pancreatic cancer cells, and the existence of CD10^+^ PSCs suggested a poor prognosis. In the present study, we found that CD10 mRNA expression continuously increased in cocultures with pancreatic cancer cells but did not decrease in PSCs migrating through Matrigel toward pancreatic cancer cells (data not shown). In contrast, CD271^+^ PSCs existed near pancreatic tumors, but were separate from pancreatic cancer cells, rather than adjacent, and the existence of CD271^+^ PSCs was an independent factor for good prognosis. These findings suggest that CD271^+^ PSCs may play a defensive role in pancreatic carcinogenesis and/or progression of pancreatic cancer, and that long-term interactions with cancer cells may reduce the number of CD271^+^ PSCs. However, CD271^+^ PSCs were too difficult to isolate after primary cultures. Therefore, we were unable to evaluate directly the function of CD271^+^ PSCs, and further investigations are required.

In conclusion, CD271^+^ PSCs existed near pancreatic cancer tumors, and CD271 expression correlated with a better prognosis of human pancreatic cancer. A short interaction with cancer cells transiently increased the expression of CD271 in PSCs, while a long interaction with cancer cells decreased CD271 expression in PSCs. CD271^+^ PSCs appeared during the early stages of cancer–stroma interactions. Taken together, the present findings suggest that the CD271^+^ subpopulation of PSCs may play a role in the resistance to pancreatic carcinogenesis and progression of pancreatic cancer.

## Supporting Information

Table S1 Clinicopathological characteristics of the patients (n = 105).
(DOCX)

Table S2 Univariate survival analyses for conventional prognostic factors and stromal CD271 expression (n = 105).
(DOCX)
