# Supplementary material for: CD271+ Subpopulation of Pancreatic Stellate Cells Correlates with Prognosis of Pancreatic Cancer and Is Regulated by Interaction with Cancer Cells
Source: PLoS One. 2012 Dec 27;7(12):e52682. doi: 10.1371/journal.pone.0052682 (PMC3531333; doi:10.1371/journal.pone.0052682)
Supplement: Table S1 — Clinicopathological characteristics of the patients (n = 105). (DOCX) [file pone.0052682.s001.docx]

**Table S1.** Clinicopathological characteristics of the patients (n=105).

| Median age | | 65 years (range, 36–86 years) |
| --- | --- | --- |
| Sex | Male | 65 (61.9%) |
|  | Female | 40 (38.1%) |
| Histological diagnosis | Invasive ductal adenocarcinoma | 102 (97.1%) |
|  | Adenosquamous carcinoma | 3 (2.9%) |
| pT category | pT1 | 10 (9.5%) |
|  | pT2 | 4 (3.8%) |
|  | pT3 | 88 (83.8%) |
|  | pT4 | 3 (2.9%) |
| pN category | pN0 | 29 (27.6%) |
|  | pN1 | 76 (72.4%) |
| UICC stage | I | 11 (10.5%) |
|  | II | 91 (86.7%) |
|  | III | 3 (2.9%) |
|  | IV | 0 (0.0%) |
| Histological grade | G1 | 13 (12.4%) |
|  | G2 | 37 (35.2%) |
|  | G3 | 50 (47.6%) |
|  | others | 5 (4.8%) |
| Pathological margin | Negative | 75 (71.4%) |
|  | Positive | 30 (28.6%) |

UICC, Union for International Cancer Control.
