# Supplementary material for: CD271+ Subpopulation of Pancreatic Stellate Cells Correlates with Prognosis of Pancreatic Cancer and Is Regulated by Interaction with Cancer Cells
Source: PLoS One. 2012 Dec 27;7(12):e52682. doi: 10.1371/journal.pone.0052682 (PMC3531333; doi:10.1371/journal.pone.0052682)
Supplement: Table S2 — Univariate survival analyses for conventional prognostic factors and stromal CD271 expression (n = 105). (DOCX) [file pone.0052682.s002.docx]

**Table S2.** Univariate survival analyses for conventional prognostic factors and stromal CD271 expression (n=105).

| Characteristics | | No. of cases | Median survival time (months) | 5-Year survival rate | P value |
| --- | --- | --- | --- | --- | --- |
| CD271 positivity | negative | 76 | 18.5 | 28.6% | 0.0040 |
|  | positive | 29 | 62.0 | 64.0% |  |
| Age | < 65 | 51 | 21.5 | 43.0% | 0.7421 |
|  | ≥ 65 | 54 | 25.0 | 33.2% |  |
| pT category | pT1 / pT2 | 14 | 62.0 | 83.9% | 0.0349 |
|  | pT3 / pT4 | 91 | 22.5 | 32.6% |  |
| Histolpgic grade | G1 | 13 | 17.0 | 46.2% | 0.9259 |
|  | G2 | 37 | 26.5 | 37.0% |  |
|  | G3 | 50 | 22.5 | 43.0% |  |
|  | others | 5 | 15.5 | 25.0% |  |
| Lymph node metastasis | No | 29 | 62.0 | 68.6% | 0.0003 |
|  | Yes | 76 | 17.5 | 27.6% |  |
| UICC stage | I | 11 |  | 100.0% | 0.0209 |
|  | II | 91 | 18.5 | 32.6% |  |
|  | III / IV | 3 | 24.0 | 0.0% |  |
| Perilymphatic invasion | No | 30 | 62.0 | 63.0% | 0.0093 |
|  | Yes | 75 | 18.5 | 30.3% |  |
| Perivascular invasion | No | 44 | 62.0 | 57.5% | 0.0025 |
|  | Yes | 61 | 16.5 | 27.4% |  |
| Perineural invasion | No | 19 | 24.5 | 45.2% | 0.7833 |
|  | Yes | 86 | 23.5 | 36.5% |  |
| Pathological margin | Negative | 75 | 62.0 | 54.2% | <0.0001 |
|  | Positive | 30 | 12.5 | 5.8% |  |
